# Fibroblast Growth Factor Receptor 1 Amplification in Non-Small Cell Lung Cancer by Quantitative Real-Time PCR

**DOI:** 10.1371/journal.pone.0079820

**Published:** 2013-11-08

**Authors:** Shirish M. Gadgeel, Wei Chen, Michele L. Cote, Aliccia Bollig-Fischer, Susan Land, Ann G. Schwartz, Gerold Bepler

**Affiliations:** Karmanos Cancer Institute & Department of Oncology, Wayne State University, Detroit, Michigan, United States of America; Winship Cancer Institute of Emory University, United States of America

## Abstract

**Introduction:**

Amplification of the fibroblast growth factor receptor 1 (FGFR1) gene has been described in tumors of non-small-cell lung cancer (NSCLC) patients. Prior reports showed conflicting rates of amplification frequency and clinical relevance.

**Materials and Methods:**

We developed a reliable real-time quantitative PCR assay to assess the frequency of FGFR1 amplification and assessed the optimal cutoff level of amplification for clinical application.

**Results:**

In a training cohort of 203 NSCLCs, we established that a 3.5-fold amplification optimally divided patients into groups with different survival rates with a clear threshold level. Those with FGFR1 amplification levels above 3.5-fold had an inferior survival. These data were confirmed in a validation cohort of 142 NSCLC. After adjusting for age, sex, performance status, stage, and histology, patients with FGFR1 amplification levels above 3.5 fold had a hazard ratio of 2.91 (95% CI- 1.14, 7.41; pvalue-0.025) for death in the validation cohort. The rates of FGFR1 amplification using the cutoff level of 3.5 were 5.1% in squamous cell and 4.1% in adenocarcinomas. There was a non-significant trend towards higher amplifications rates in heavy smokers (> 15 pack-years of cigarette consumption) as compared to light smokers.

**Discussion:**

Our data suggest that a 3.5-fold amplification of FGFR1 is of clinical importance in NSCLC. Our cutpoint analysis showed a clear threshold effect for the impact of FGFR1 amplification on patients’ survival, which can be used as an initial guide for patient selection in trials assessing efficacy of novel FGFR inhibitors.

## Introduction

A paradigm shift in the management of non-small-cell lung cancer (NSCLC) patients has been the identification of therapeutically actionable ‘driver’ genetic alterations [1]. The number of these genetic alterations is steadily increasing [2]. However, most alterations have been identified in adenocarcinomas of the lung. Therefore, the therapeutic impact of this paradigm shift has been minimal for patients with squamous cell carcinoma of the lung.

Recently, amplification of the fibroblast growth factor receptor 1 (FGFR1) gene has been described as an oncogenic alteration in a subgroup of squamous cell carcinomas [3,4]. FGFR1 belongs to the FGFR family of receptors and is involved in inflammation, wound healing and embryonic development. Since the FGFR family of receptors appears to have a role in many cancers, several inhibitors of FGFR are being developed [5]. A single case report has shown that the FGFR inhibitor BGJ398 did demonstrate partial response in a patient with squamous cell lung carcinoma whose tumor was amplified for FGFR1 [6].

An essential aspect for therapeutic targeting of genetic alterations in lung cancer is the rapid, specific, and precise identification of alterations in patient samples. Many investigators have utilized fluorescent in situ hybridization (FISH) to detect FGFR1 amplification [7-11]. The definition of FGFR1 amplification has varied among the various reports. In addition, FISH analysis is laborious, technically complex, and reader dependent. These characteristics limit its clinical applicability. We developed a quantitative, real-time PCR test, which is easier to perform and robust in its interpretation, to evaluate NSCLCs for FGFR1 amplification and assessed the clinical characteristics and prognostic relevance of this genetic alteration. Our ultimate goal is to be able to identify NSCLC patients that can derive clinical benefit from FGFR1 inhibitors utilizing this PCR based test.

## Patients and Methods

### Specimen collection and outcomes data

Collection of biospecimens and outcomes data complied with the Helsinki Declaration and was approved by the Wayne State University School of Medicine Institutional Review Board. Tumor materials used in this research were from patients who provided written informed consent. Fresh-frozen tumor specimens were collected prospectively from patients who underwent a surgical resection for diagnosed or suspected lung cancer. All patients who were candidates for surgical resection of their lung cancer, either biopsy proven or suspected, were consented for specimen collection. Only patients whose tumors were confirmed to be NSCLC were included in this analysis. Specimens from patients with a final diagnosis of small cell carcinoma (N=16) or a small cell component of NSCLC (N = 2), carcinoid tumors (N = 6), or mesothelioma (N = 3) were excluded. Specimens were kept frozen at -80°C in aliquots of approximately 0.1 g. Specimen procurement procedures were developed to reduce the resection to freezing time interval to less than 30 min. The quality of extracted analytes was assured by performing integrity analysis. The overall procurement period ranged from 1985 to 2001. Formalin-fixed and paraffin-embedded specimens were reviewed to verify diagnosis and to determine tumor cell content. Specimens were uniquely identified by laboratory numbers that allowed cross-referencing with clinical data from the tumor registry and chart review without disclosure of patient identity. Demographic and clinical outcomes data collected included the dates of birth, diagnosis (defined as the date of first pathologic verification of malignancy), surgical resection (same as the date of specimen procurement), and date of last follow-up or death; sex, race, tumor histology, pathological and clinical tumor stage (version 6), performance status, weight loss (defined as >5% during the 3 months proceeding surgery), and self-reported smoking history (defined as life-time never smoker for those who had smoked <100 cigarettes, former smoker for those who had quit cigarette smoking for more than one year, and smoker for all others). One of the patients included in this analysis had preoperative chemotherapy and radiation and one had preoperative radiation. None of the patients had adjuvant therapy. A standardized protocol for patients’ follow-up evaluation was not specified.

Specimens were divided into a training and non-overlapping validation cohort. The details of the validation cohort have previously been reported [12].

### DNA extraction and quantitative PCR analysis

Specimens (~100 mg) were pulverized with separate, sterilized, and frozen mortar and pestles. DNA was extracted using resin-based or phenol-based extraction techniques, and specimens were aliquoted and stored refrigerated. DNA quantity and quality was assessed using the Quantifiler^®^ human DNA quantification kit (Life Technologies, Carlsbad, CA), an approach to quantify amplifiable DNA present in a sample using a real-time PCR TaqMan^®^ assay that targets a 62 base pair sequence in the human telomerase gene. A standard curve of known DNA concentrations was also analyzed for comparison and quantitation of each DNA sample. The real-time fluorescent TaqMan^®^ reaction described by Hied, et al, relies on a hybridization probe labeled with two distinct dyes, a reporter dye (FAM) and a non-fluorescent quencher dye [13]. When the probe is intact and not extended, the fluorescent emission of the reporter dye is absorbed by the quenching dye. When the probe is specifically annealed to the corresponding DNA target sequence, during the extension phase of the PCR the probe is cleaved by the 5'-3' nucleolytic activity of the DNA polymerase. On cleavage of the probe, the quenching dye is liberated from the complex resulting in an increase of the reporter dye fluorescent emission spectra.

Copy number analysis for the human FGFR1 gene was performed by real-time PCR using two independent TaqMan^®^ assays (Life Technologies, Carlsbad, CA) specific for exon 15 (catalogue number Hs02702320; targeting Chr.8:38274932 on NCBI build 37, overlapping the intron 14 - exon 15 boundary within the kinase domain region) and exon 19 (catalogue number Hs00237051; targeting Chr.8:38271828 on NCBI build 37, overlapping the intron 18 - exon 19 boundary within the kinase domain region). Relative quantitation of gene copy number in cancer samples was done by the Livak (2^-ΔΔCT^) method using CEPH 1347-02 control DNA and RPLPO as the reference gene (catalogue number 4326314E), both from Life Technologies [14]. The average value of 3 replicates was calculated and used as gene amplification level.

DNA quantitation and copy number TaqMan^®^ assays were run and emission spectra data collected using an Applied Biosystems^®^ 7900 Real-Time PCR System (Life Technologies).

### Statistical analysis

The optimal FGFR1 amplification threshold for separating patients with long and short overall survival (OS) was first determined in a training cohort and then confirmed in an independent and external validation cohort. The primary endpoint was OS, defined as time interval from date of diagnosis to death from any cause. Patients who were alive or lost to follow-up were censored at the date last seen alive. Patients’ descriptive characteristics at baseline were reported for the training and validation cohorts. Due to over fitting issue in identifying the outcome related marker cutoff value, a ten-fold cross validation within the training cohort was used. Briefly, the training cohort was randomly partitioned into 10 portions. Each time, one portion was set aside and the remaining nine portions were used to identify the best cutoff, defined as the one that reached the most significant log-rank test on the association between OS and the group assignment (amplified or non-amplified) among all possible cutoffs. The identified best cutoff was then used to obtain a group assignment for each of the patients in the set-aside portion, which was not used for determining the cutoff. This process was repeated for each of the 10 portions, until all the patients in the training cohort had a group assignment. A log-rank test was then performed on the entire training cohort. We repeated this process 1000 times to obtain a distribution of best cutoffs. The cutoff with the highest frequency was the final cutoff that was tested in the validation cohort. 

The log-rank test was used for confirming the cutoff in the validation cohort. A multivariate Cox model was used for evaluating the independent prognostic role of FGFR1 amplification adjusted for age, sex, performance status (PS), stage, and histologic subtype. The PS was missing 10% in the validation data, which would reduce the sample size and power if only completed observations were analyzed. Since we believe that PS is completely missing at random in this clinical set, we modified the multiple imputation method proposed originally by Rubin and implemented it in the Cox model for validation data (15,16). Briefly, we only imputed the missing values two times (M=2). One with all the missing PS imputed with 0, and the other with all the missing PS imputed with 0, the only other choice for the dichotomized PS. This model free multiple imputation approach fits our needs to estimate the effect of our variable of interest, the amplified or non-amplified FGFR1, under the extreme circumstance of covariates. The estimated standard error is given by Rubin’s formula [15,16]. All *p*-values were 2-sided with a significance level of 0.05. All calculations were performed with R Version 2.14.0 [17]

## Results

### Clinical characteristics of training and validation cohorts

DNA of sufficient quantity and quality was obtained from 347 tumor specimens from patients with NSCLC. OS data were available on 345 patients, whose baseline characteristics are listed in Table 1. The median age was 66 years, 66% were male, and the median cigarette consumption was 50 pack-years (PY). The majority (66%) of patients had stage I disease, and adenocarcinomas (49%) and squamous cell carcinomas (39%) were the major histologic categories. The baseline characteristics of the training and validation cohorts were similar, although the validation cohort had more non-whites (p<0.001), worse PS (p<0.001) and slightly greater median pack-years of smoking (p=0.033) compared to the training cohort.

**Table 1:** Patient characteristics.

**Variable**	**Training Cohort**	**Validation Cohort**	P Value*
	**(n=203)**	**(n=142)**	
**Age** Median (range)	66.2 (35.0 - 83.8)	65.2 (25.8 - 81.9)	0.144
**Sex**			
Male	133 (66%)	93 (65%)	1.000
Female	70 (34%)	49 (35%)	
**Race**			
White	197 (97%)	125 (88%)	<0.001
African American	3 (1%)	15 (11%)	
Other	3 (1%)	2 (1%)	
**Histology**			
Adenocarcinoma	98 (48%)	71 (50%)	0.108
Squamous	79 (39%)	57 (40%)	
Large cell	15 (7%)	13 (9%)	
Other	11 (5%)	1 (1%)	
**Stage**			
IA	56 (28%)	42 (30%)	0.857
IB	83 (41%)	48 (34%)	
IIA	7 (3%)	6 (4%)	
IIB	32 (16%)	27 (19%)	
IIIA	16 (8%)	12 (8%)	
IIIB	2 (1%)	3 (2%)	
IV	6 (3%)	4 (3%)	
**Performance Status**			
0	147 (72%)	29 (20%)	<0.001
1	44 (22%)	79 (56%)	
2	1 (<1%)	20 (14%)	
Missing Data	11 (5%)	14 (10%)	
**Weight Loss**			
Absent	162 (80%)	117 (82%)	0.049
Present	28 (14%)	9 (6%)	
Missing Data	13 (6%)	16 (11%)	
**Smoking History**			0.247**
Never smoker	10 (5%)	11 (8%)	
Light smoker (<15 PY)	13 (6%)	3 (2%)	
Heavy smoker (>15 PY)	163 (80%)	106 (75%)	
Missing Data	16 (8%)	22 (15%)	

* Fisher’s exact test for categorical variables and Wilcoxon’s rank sum test for continuous variables age and pack-years.

** Fisher’s exact test between never and ever smokers.

### Concordance between the copy number variations (CNV) of exon 15 and exon 19 of the FGFR1 gene

**Figure 1:**
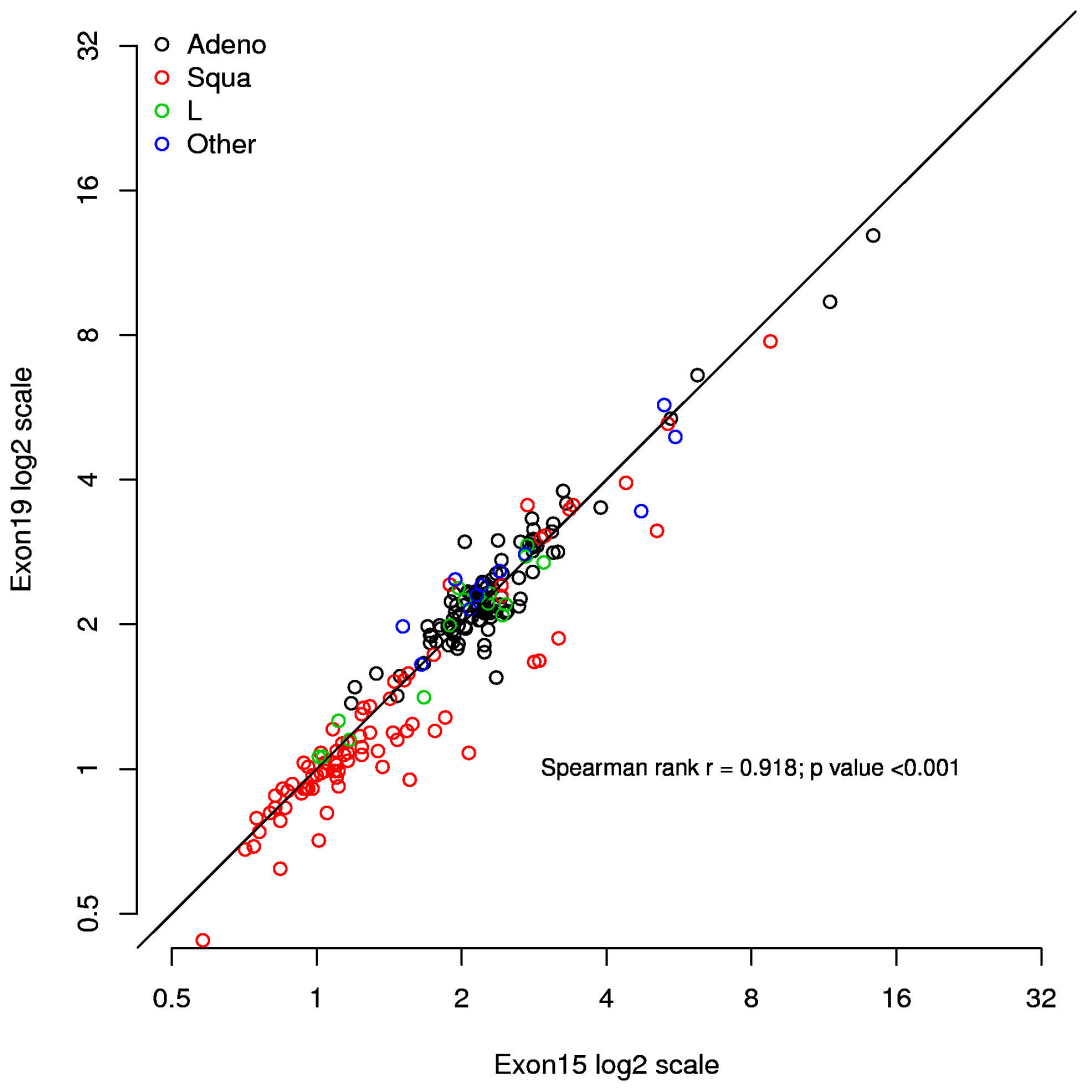
Scatter plot of exon 15 and 19 CNVs in the training cohort.

We first assessed concordance between exon 15 and exon 19 CNV for the FGFR1 gene using our newly developed real-time PCR assay in the training cohort of 203 NSCLC patients. Exon 15 CNVs ranged from 0.58 - 14.32, exon 19 CNVs ranged from 0.44 - 12.89, and they were highly correlated (Spearman rank r = 0.918, p < 0.001) (Figure 1).

### Optimal cut-point determination in the training cohort

**Figure 2:**
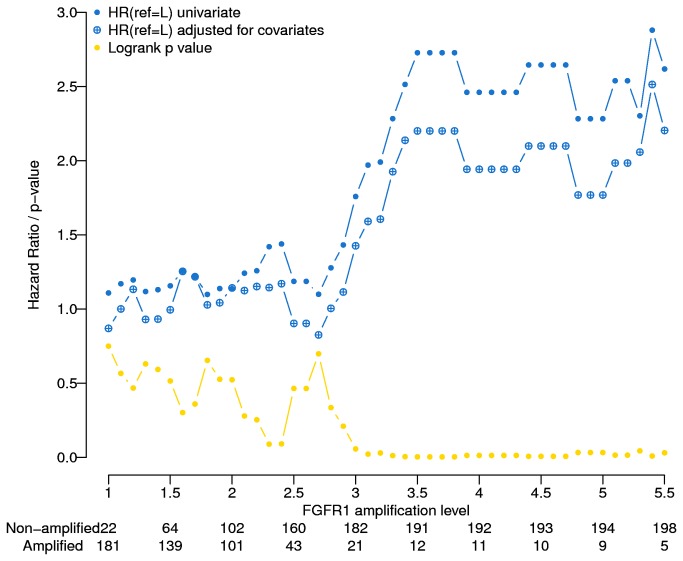
Exon 15 cutoff points and hazard ratio for death in the training cohort.

We then determined the level of the exon 15 CNV that produced the optimal separation of patients in the training cohort into groups with short and long OS. Using a CNV threshold of 3.50 resulted in the best separation, with 191 patients having levels less than 3.50 and 12 (5.9%) patients greater than 3.50. Patients with a high CNV had significantly greater likelihood of death compared to those with CNVs below the threshold (HR = 2.19, 95% CI: 1.02, 4.75; *p* = 0.045; Figure 2). Adjusting for the covariates age, sex, performance status, stage, and histology yielded the same cutpoint of 3.50 (Figure 2).

**Figure 3:**
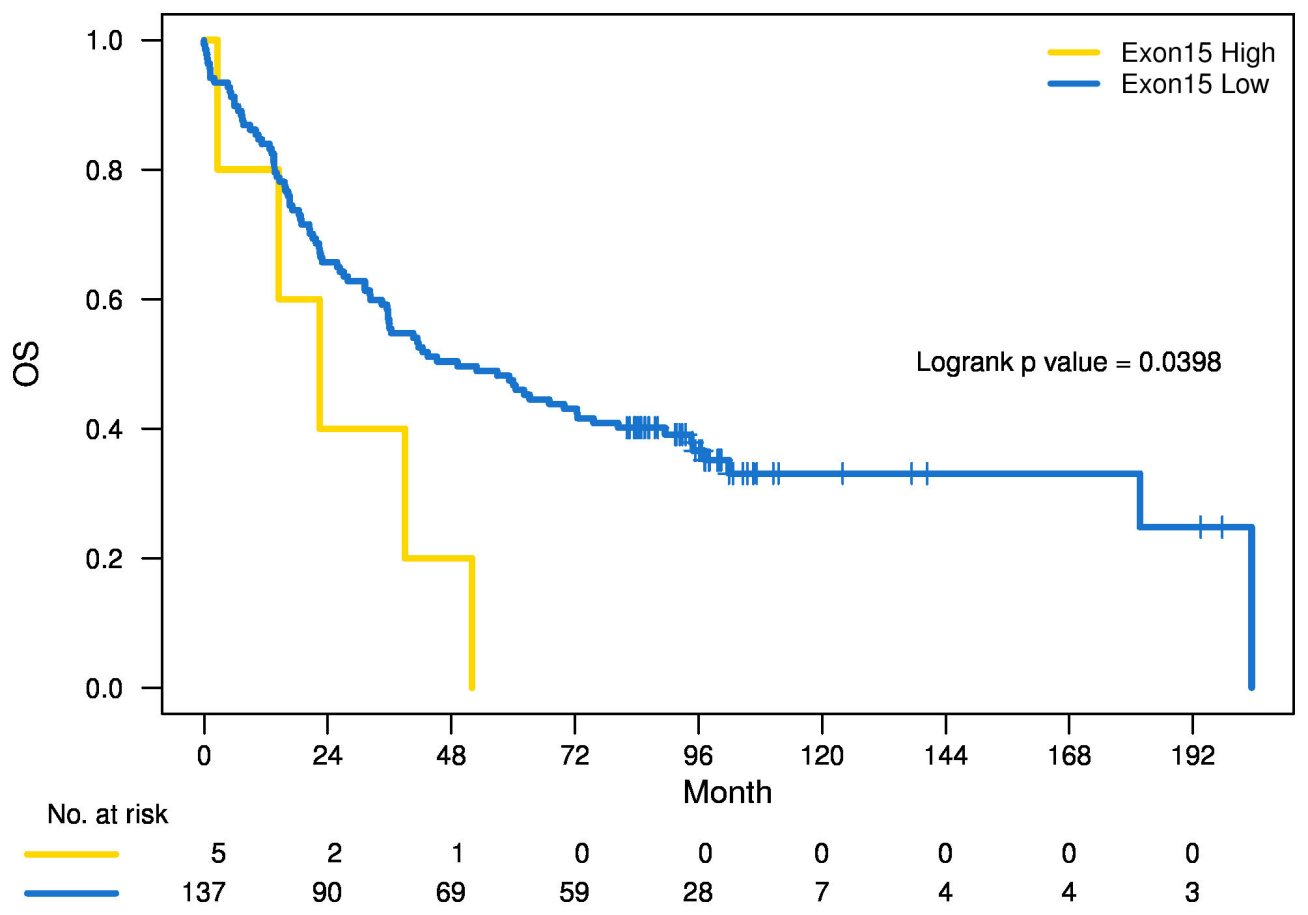
Kaplan-Meier overall survival estimates in the validation cohort.

### Results in the validation cohort

To confirm the OS impact of the CNV threshold of 3.50, we analyzed the validation cohort of 142 patients (exon 15 CNV range 0.69 - 46.54). Five patients (3.5%) had high levels, and 137 had low levels. We performed a multivariate Cox regression analysis adjusting for the same covariates and were able to confirm that patients with high CNVs and a hazard ratio for death of 2.91 (95% CI: 1.14, 7.41; *p* = 0.025; Table 2). Kaplan-Meier survival estimates demonstrated longer OS for patients with low CNVs compared to those with high CNVs (*p* = 0.0398; Figure 3).

**Table 2:** Multivariate Cox model in the validation cohort (N = 142).

**Variable**	**Hazard Ratio (95% CI)**	***p*-Value**
**FGFR1 CNV value**		
<3.50 (n = 137)	Reference	0.025
>3.50 (n = 5)	2.91 (1.14 - 7.41)	
**Stage**		
I (n = 90)	Reference	0.005
>I (n = 52)	1.90 (1.22 - 2.96)	
**Age** (as continuous variable)	1.01 (0.99 - 1.03)	0.35
**Sex**		
Male (n = 93)	Reference	0.11
Female (n = 49)	0.68 (0.42 - 1.09)	
**Performance Status**		
0 (n = 29)	Reference	0.15
>0 (n = 113)	1.57 (0.86 - 2.88)	
**Histology**		
Adenocarcinoma (n = 71)	Reference	
Squamous (n = 57)	1.08 (0.69 - 1.68)	0.75
Large cell (n = 13)	1.16 (0.51 - 2.62)	0.73
Other (n = 1)	NA	NA

### Frequency and characteristics of patients with FGFR1 amplification

We then combined the training and validation cohorts and analyzed the rate of patients with FGFR1 amplification (defined as a CNV >3.50) by baseline characteristics (Table 3). The overall rate of FGFR1 amplifications was 4.9% (17/345). FGFR1 amplification was more frequent in men (7%) compared to women (2%), *p* = 0.064. There was no significant difference in the rate of FGFR1 amplification between adenocarcinomas (4.1%) and squamous cell carcinomas (5.1%). We did not find a statistically significant difference for FGFR1 amplification between tumors of patients with ‘light’ (<15 PY) and ‘heavy’ (≥ 15 PY) smoking histories (3% versus 6%), although there was a trend towards a higher rate among ‘heavy’ smokers. The number of patients who were life-time never smokers compared to ever smokers with and without FGFR1 amplification was too low for a statistically meaningful comparison.

**Table 3:** Distribution of FGFR1 amplification in all patients (N = 345).

**Variable**	**FGFR1 CNV >3.50**	**FGFR1 CNV <3.50**	*p*-Value*
**Sex**			
Male	15 (7%)	211 (93%)	0.06
Female	2 (2%)	117 (98%)	
**Race**			
White	17 (5%)	305 (95%)	1
African American	0 (0%)	18 (100%)	
Other	0 (0%)	5 (100%)	
**Smoking History**			
<15 PY	1 (3%)	36 (97%)	0.74
>15 PY	15 (6%)	255 (94%)	
Missing Data	1 (3%)	37 (97%)	
**Histology**			
Adenocarcinoma	7 (4%)	162 (96%)	0.04
Squamous	7 (5%)	129 (95%)	
Large cell	0 (0%)	28 (100%)	
Other	3 (25%)	9 (75%)	
**Stage**			
I	9 (4%)	220 (96%)	0.29
>I	8 (7%)	108 (93%)	
**Performance Status**			
0	9 (5%)	167 (95%)	0.59
>0	6 (4%)	138 (96%)	
Missing Data	2 (8%)	23 (92%)	

* Fisher’s exact test

## Discussion

The ability to identify and target oncogenic alterations in NSCLCs has been a major advance in the management of patients. An important aspect of translating these molecular alterations into clinical practice is to develop assays that can quickly and reliably identify specific aberrations in clinical specimens. Our results suggest that the quantitative real-time PCR assay developed can identify tumors with clinically significant FGFR1 amplification.

FGFR signaling is activated in many cancers including oral squamous cell, ovarian, bladder, and breast cancers [5,18-22]. Weiss et al. were among the first to report that the chromosomal 8p12 segment, which includes the FGFR1 gene, is amplified in 9.7% of squamous cell carcinomas of the lung [3]. In addition, the authors reported that patients with tumoral FGFR1 amplification tended to have inferior survival, albeit non-significant. Their analysis included 77 adenocarcinomas, and they found FGFR1 amplification only in 1% of the patients. They also analyzed an independent set of 153 squamous cell carcinomas utilizing an 8p12-specific FISH probe and detected FGFR1 amplification (defined as >9 copies in an unspecified fraction of cells) in 22% of patients. Dutt et al. assessed the 8p11-12 segments in over 600 lung cancers utilizing SNP array technology and found it amplified (defined as 3.25 copy number variation) in 6% of NSCLCs, 21% (12/57) in squamous cell and 3.4% (20/588) in adenocarcinomas [4]. In addition, they showed that proliferation of NSCLC cell lines with amplification appeared to be dependent upon FGFR1 pathway activation.

Several other investigators have analyzed clinical samples of NSCLCs for presence of FGFR1 amplification by FISH [7-11]. There is considerable variability in defining a positive result in these reports. However, all reported a significantly higher rate in squamous cell carcinomas compared to adenocarcinomas. Some reports found higher rates in men compared to women, and some reports found FGFR1 amplification prognostic of inferior survival [8,9]. The data regarding the prognostic impact of FGFR1 amplification varies among the different studies; with some studies showing a worse survival [3,7] and others showing no difference in outcomes [8]. 

The variability of FGFR1 amplification rates as determined by FISH is related to differences in the definition of a positive result and in interpretation of results. For instance, in an analysis of 420 lung cancers, a group of investigators from Germany defined a tumor as highly amplified if the FGFR1/centromere ratio was ≥ 2.0, the average number of FGFR1 signals per tumor cell nucleus was ≥6, or if large clusters ( ≥ 15 FGFR1 signals) were found in ≥ 10% of the counted nuclei. With these criteria, they reported an FGFR1 amplification rate of 16% [11].

FISH analysis is laborious, technically complex, and reader dependent. All are characteristics that limit its clinical applicability. We therefore designed an assay that evaluates FGFR1 gene amplification by real-time quantitative PCR, which is technically less complex, automated, quantitative, and independent of reader interpretation. Our investigation revealed that a 3.5-fold amplification of the FGFR1 gene was prognostic of inferior survival. In addition, our optimal cutpoint analysis suggests that there is a clear threshold (Fig. 2) for the interaction between gene amplification and survival; i.e., the hazard ratio remains approximately 1.0 for amplification levels below 3.0-fold and stays at approximately 2.5-fold for levels above 3.5-fold. Using this cutpoint, the rate of FGFR1 amplified tumors was 5.1% in squamous cell carcinomas. This rate is lower than those reported in other manuscripts because of a higher cutpoint level. For instance, had we set the cutpoint to a 2.0-fold amplification, 19% (26/136) of squamous cell carcinomas would have been positive. Although this amplification rate is more consistent with prior reports, we did not opt to use this level because it lacks prognostic clinical utility. Interestingly, The Cancer Genome Atlas Research (TCGA) network recently reported a comprehensive analysis of genomic and epigenomic alterations in 178 squamous cell carcinomas of the lung and found that the rate of FGFR1 amplification was 7% [23]. Thus, our results are similar to the TCGA findings.

We did not find a significant difference in the rate of FGFR1 amplification between squamous cell and adenocarcinomas as previously reported [3,4]. This may be explained by selection bias, differences in technologies and cutpoint definitions, variability in histologic interpretation, or the impact of smoking history. Some reports have suggested that the rate of FGFR1 amplification is higher in smokers, and amplifications may not be observed in never smokers [7]. We observed a trend towards a higher rate of FGFR1 amplification in ‘heavy’ smokers as compared to ‘light’ smokers. It is possible that the rate of FGFR1 amplification is related to smoking history, specifically prolonged smoking, which may account for the perceived association with squamous cell carcinoma, since it is this subtype that is most closely associated with cigarette consumption. Our rate of 4.1% FGFR1 amplification in adenocarcinomas is similar to the rate of amplification detected in other publications [3,11].

The identification of FGFR as a potential ‘driver’ of cancers has spurred interest in the development of pathway inhibitors. Ongoing trials are evaluating the role of FGFR inhibitors in tumors with FGFR1 activation, including squamous cell carcinoma of the lung. Recently, Wolf et al. reported a confirmed response to BGJ398, an FGFR inhibitor, in a squamous cell carcinoma patient whose tumor had an FGFR1/CEP8 ratio of 2.6 by FISH [6]. Whether our quantitative PCR assay can be used to predict responses to FGFR inhibitors remains to be determined, as does the optimal cutpoint to predict response. However, our data suggest that a 3.5-fold amplification is a reasonable preliminary starting point.

In conclusion, we have developed a quantitative real-time-PCR assay for quick and reliable determination of FGFR1 amplification in tumor specimens. Our data suggest that a 3.5-fold amplification is of clinical importance in NSCLC since patients with higher levels have shorter survival than those with lower levels. The frequency of amplifications above this level is 5.1% in squamous cell carcinomas and 4.1% in adenocarcinomas. Our cutpoint analysis suggests that there is a clear threshold effect for the impact of FGFR1 amplification on patients’ survival.
